# Two Advanced Cryogenic Procedures for Improving *Stevia rebaudiana* (Bertoni) Cryopreservation

**DOI:** 10.3390/plants10020277

**Published:** 2021-01-31

**Authors:** Carla Benelli, Lara S. O. Carvalho, Soumaya EL merzougui, Raffaella Petruccelli

**Affiliations:** 1 Institute of BioEconomy, National Research Council (CNR/IBE), 50019 Sesto Fiorentino, Florence, Italy; raffaella.petruccelli@ibe.cnr.it; 2 Department of Biology, Federal University of Lavras, Lavras 3037, Brazil; lcarvalho470@gmail.com; 3 Laboratory of Biotechnology and Valorization of Natural Resources (LBVRN), Faculty of Sciences, Ibn Zohr University, 8106 Agadir, Morocco; soumaya.merzougui@gmail.com

**Keywords:** apical shoot tips, axillary shoot tips, droplet-vitrification, long-term conservation, PVS2 vitrification, V cryo-plate

## Abstract

Cryopreservation is a useful tool for the long-term storage of plant genetic resources, and different cryogenic procedures have recently been developed. The present study focused on the use of the Droplet-vitrification (DV) and V cryo-plate protocol for the cryopreservation of *Stevia rebaudiana* in vitro-derived apical shoot tips and axillary shoot tips. A preliminary test showed that 90 and 120 min PVS2 (Plant Vitrification Solution 2) treatment significantly reduced the regrowth of the explants before immersion in liquid nitrogen (LN). For both procedures tested, the best osmoprotective condition for obtaining a higher regrowth of cryopreserved explants occurred when explants were PVS2 treated for 60 min. After direct immersion in LN, thawing and plating, the highest regrowth recorded was 80% with DV and 93% with V cryo-plate. Moreover, shoot tips proved to be a more suitable material for Stevia cryopreservation. A satisfactory vegetative regrowth was observed in the subcultures following cryopreservation by DV and V cryo-plate cryogenic procedures.

## 1. Introduction

*Stevia rebaudiana* (Bertoni) is an herbaceous perennial plant of the Asteraceae family. Its leaves produce diterpene glycosides (stevioside and rebaudiosides), and as stevioside is 300-hundred-fold sweeter than sucrose, it is deemed to be a good natural sugar substitute [1,2]. In addition to its sweetening properties, it has various medicinal properties and actions. For this reason, the Stevia plant is an extremely interesting crop for breeders who select varieties with high diterpene glycosides content as well as for propagators and the target market.

*S. rebaudiana* is a self-incompatible plant and one of its limiting factors for large-scale cultivation is its poor seed germination [3]. Moreover, plants from seed propagation have a great variability in growth, maturity, and non-uniform plants, with considerable variations in the sweetening level and composition [4,5]. The recent results suggest that seed germination and stem cutting are not cost effective methods for higher biomass production, while the micropropagation can be a promising technique [3].

*In vitro* conservation and cryopreservation are unconventional biotechnological tools to preserve selected and valuable lines of *S. rebaudiana*, also taking into account the problems relating to its propagation by seed [2,3].

A protocol for in vitro conservation of *S. rebaudiana* under slow growth conditions and mass micropropagation after the storage period was developed by Zayova et al. [6], while the long-term storage, cryopreservation of shoot tips, was carried out using the vitrification method by Shatnawi et al. [7]. Cryopreservation allows the storage of plant material (i.e., seeds, shoot tips, dormant buds, zygotic, and somatic embryos and pollen) at ultra-low temperatures in liquid nitrogen (LN; −196 °C) or in the vapour phase of LN (−165 °C to −170 °C) [8] and it is becoming a widely practised method for the long-term storage of plant genetic resources [9,10,11,12]. An advantage of the cryopreservation is that plant germplasm can theoretically be kept indefinitely in very little space and at low cost, excluding the initial investment. Over the last 30 years, various cryopreservation techniques have been developed using conventional slow freezing methods [13,14,15] as well as several vitrification-based cryopreservation procedures (encapsulation-dehydration; vitrification; encapsulation-vitrification [16,17,18], and more recently, droplet-vitrification (DV) [19]. Two recent novel cryopreservation techniques have been identified and have resulted in V cryo-plate [20] and D cryo-plate [21]. The DV uses aluminum foil strips, while the most recent cryogenic procedure (D or V cryo-plate) uses aluminum cryo-plates.

The regrowth rate obtained in Stevia with the vitrification protocol by Shatnawi et al. [7] was 68%. Continuous research and technological evolution have markedly improved the cryogenic methodologies, allowing to enhance the recovery percentage of the species, as has occurred over the years, for example, in *Vitis* spp. [22,23] and potato [24,25]. The aim of this study was to assess the efficiency of the novel procedures, Droplet-vitrification and V cryo-plate, in order to improve the *S. rebaudiana* cryopreservation protocol.

## 2. Results

### 2.1. Evaluation Plant Vitrification Solution 2 (PVS2) Tolerance

**Figure 1:**
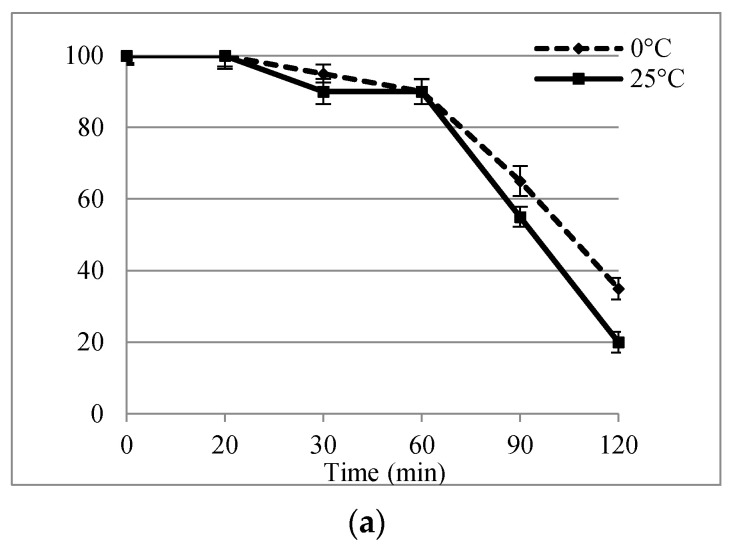

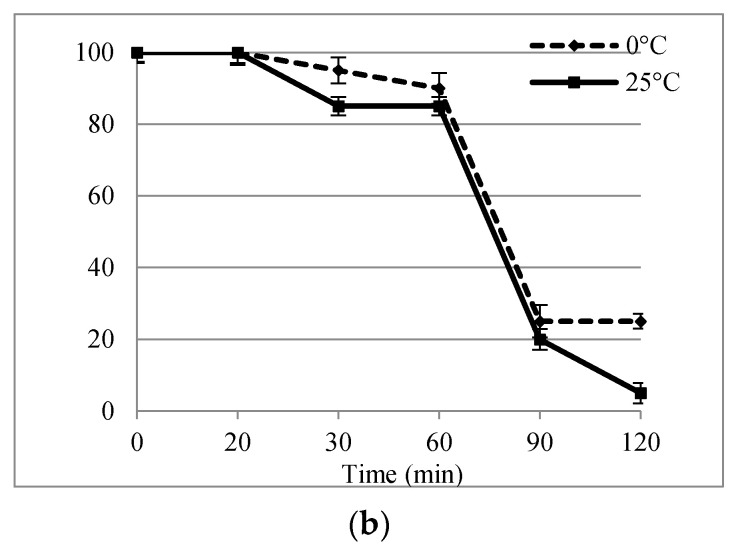
Effect of PVS2 treatment times on regrowth percentage (± SD) of *S. rebaudiana* apical shoot tips (**a**) and axillary shoot tips (**b**) at 0 °C and 25 °C, after 28 days of culture.

In this study, a preliminary experiment on apical shoot tips (AST) and axillary shoot tips (AxST) to evaluate the effect of exposure duration and optimize temperature of PVS2 treatment showed that exposure to PVS2 induced time-dependent regrowth in both of the explants assessed and that the temperature of the treatment can influence regrowth rates (Figure 1). After 28 days of culture, the best regrowth rates were obtained for the explants treated with PVS2 with exposure times ranging from 20 min to 60 min at 0 °C, and similar trends were observed for AST and AxST. AST treated with PVS2 solution for 60 min at both temperatures had 90% regrowth (Figure 1a), while in the AxST, the regrowth was 90% at 0 °C and 85% at 25 °C (Figure 1b). PVS2 treatments markedly affected regrowth potential after 60 min, when a significant drop was observed, suggesting a toxic response to long-term exposure to PVS2, furthermore regrowth was also affected by treatment temperature. As regards the longer exposure time (120 min), the regrowth AST decreased significantly up to 35% and 20% at 0° C and 25 °C, respectively, while for AxST the regrowth was significantly lower, dropping to 5% at 25 °C.

In the two cryopreservation procedures described below, the PVS2 exposure times of 20, 30, and 60 min at 0 °C will be applied, given that longer incubation times and 25 °C resulted in a low regrowth percentage even in explants without immersion into LN.

### 2.2. Droplet-Vitrification Procedure

In the DV procedure, the effect of PVS2 treatment on the explants was more evident after immersion in LN and the survival and regrowth percentage of *S. rebaudiana* depended on PVS2 exposure time (Table 1). The survival rate was lower for all assessed exposure times to PVS2 compared to controls. The post-thaw survival percentages of the cryopreserved explants observed after seven days ranged from 6 to 87% for AST and 0 to 50% for AxST, while the best survival rate was observed for apical shoot tips treated with PVS2 for 60 min (87%). As regards the axillary shoot tips, 30-min and 60-min PVS2 incubation times showed the same osmoprotection effect with 50% survival rate. From the data collected, it is evident that 20 min PVS2 duration time was not osmoprotective for the explants, as none of the axillary shoot tips survived and only 6% of apical shoot tips survived, while no regrowth was observed after 28 days. Due to the lack of shoot development, this PVS2 time will not be considered for evaluating biometric parameters in the subculture. At the end of the experiment (28 days) the regrowth rate of the explants treated with PVS for 60 min remained high, confirming a better apical shoot regeneration response (80%) with respect to axillary shoot tips (50%). The explants that grew into normal shoots were used for the subculture evaluation described below.

**Figure 2:**
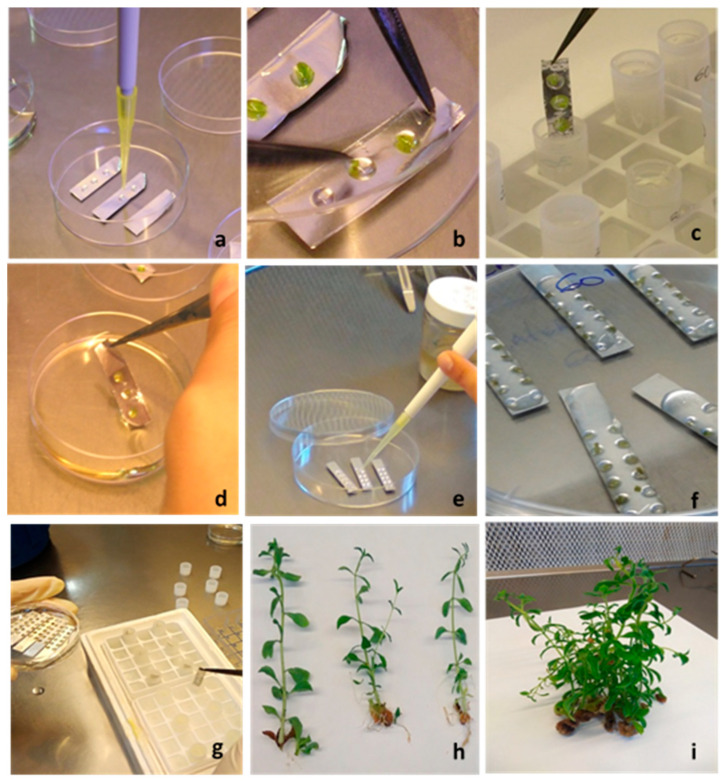
Some steps of droplet-vitrification and V cryo-plate cryopreservation procedures of *Stevia rebaudiana*. Droplet-vitrification: (**a**) PVS2 drops in aluminum foil; (**b**) explants placed into drops of PVS2; (**c**) aluminum foil strip with explants, plunged in cryovial filled with LN; (**d**) thawing of aluminum foil with explants in washing solution. V cryo-plate: (**e**) drops of sodium alginate in wells of cryo-plate; (**f**) explants into wells covered with calcium chloride solution; (**g**) cryo-plate, after PVS2 treatment, placed in cryovial filled with LN; (**h**) cryopreserved shoots with DV procedure after first subculture; (**i**) cryopreserved shoots with V cryo-plate procedure after second subculture.

Shoots derived from DV cryopreserved explants after the first subculture showed positive regrowth activity (Figure 2h), especially those obtained from initial explants treated with PVS2 + LN for 60 min, which showed similar or slightly higher values to the control (Table 2). As regards the cryopreserved shoots treated with PVS2 30 + LN, several morphological parameters considered were significantly lower than the control group and the PVS2 60 + LN treatment.

### 2.3. V Cryo-Plate Procedure

The V cryo-plate procedure proved to be effective for apical shoot tips and axillary shoot tips of *S. rebaudiana*. High viability was obtained except for the explants processed with PVS2 for 20 min followed by LN exposure. In Table 3 we reported the survival and regrowth percentages after 7 and 28 days of culture post thawing. AST gave the best response to the PVS2 treatments for cryopreservation while the AxST were more sensitive, showing lower survival and regrowth ability. In particular, treatment with PVS2 for 60 min provided better cryo-tolerance capability for the explants immersed in LN and showed the highest regrowth rate at 28 days of culture (93% AST and 67% AxST). Since minimal or no regrowth was observed from explants treated with PVS2 for 20 min + LN, this PVS2 time was not considered for evaluating biometric parameters in the subcultures.

In the first subculture, the shoots from cryopreserved AST showed a more active growth than the shoots from cryopreserved AxST (Table 4). Overall, no significant differences were observed among treatments and with the control, except for the shoot length in AST derived from PVS2 30 min + LN, which had lower values. After 28 days all of the shoots were subcultured into fresh proliferation medium.

During the second subculture, the differences between the V cryo-plate cryopreserved shoots derived from AST and AxST explants incubated in both PVS2 treatments disappeared, for all of the observed parameters (Table 5). However, it is evident that the shoots from AST treated for 30 min and 60 min + LN, even if not significantly different, were longer and produced a greater number of shoots per explant than those of the control, showing good multiplication capacity (Figure 2i).

In this study, phenotype biometric examination during in vitro subcultures, in both DV and V cryo-plate, did not reveal any morphological abnormalities compared with the control plants.

## 3. Discussion

In recent years, various biotechnological approaches have been used for conserving endangered and medicinal species, providing conservation of pathogen free plant and biodiversity. Their preservation is essential for plant breeding programs, for maintaining biodiversity, and for utilization as resource of compounds to the medicinal, food, and crop protection industries [26].

A protocol for in vitro conservation of *S. rebaudiana* under slow growth conditions and mass micropropagation after six months of storage without subculturing, was developed by Zayova et al. [6], while cryopreservation to enable the long-term storage of shoot tips was carried out using a vitrification method [7]. Several innovative procedures have recently been implemented for cryopreservation in order to improve the explant physiological state, pre-treatment conditions, time and conditions of the cryoprotectant treatments, increase the cooling and warming rates, and the recovery medium to achieve successful regrowth [12,27,28,29,30]. Droplet-vitrification and V cryo-plate are the recent cryogenic procedures available and, in this study, both methods have been applied and evaluated on *S. rebaudiana* apical shoot tips and axillary shoot tips.

In order to implement an efficient cryopreservation protocol, it is essential that plant cells can be cooled in LN and recovered without causing cell damage to maintain the cell viability. The main cause of cell injury is the transition of water into ice crystals during the cooling process [31]. To avoid the formation of ice crystals, cells and tissues have to be adequately dehydrated and/or exposed to cryoprotectant solutions, before immersing them in LN. PVS2 is the most widely used cryoprotectant solution with successful on vitrification-based cryopreservation. It is also vital to carefully control dehydration step and prevent injury by chemical toxicity or excessive osmotic stress during PVS treatment. Possible toxic effects of the PVS2 and potential damage that may occur during vitrification were studied in various species [32,33,34]. Our findings suggest that 90 min and 120 min of PVS2 treatment, both at 0 °C and 25 °C, adversely affected the *S. rebaudiana* explants, which led to a significant decrease in the apical shoot tips regrowth percentage and axillary shoot tips, thus demonstrating that explants do not tolerate prolonged exposure to this solution. While short exposure duration to PVS2 (20 min) was ineffective in achieving osmoprotection on the tissues; 30 min and 60 min at 0 °C proved to be the best treatments for achieving satisfactory regrowth of shoots tips and nodal segments following both DV and V cryo-plate procedures. This is in line with the results reported for shoot tips of *S*. *rebaudiana*, which showed 68% of regrowth when subjected to the PVS2 solution for 60 min at 0 °C using a vitrification technique [7] and is also in accordance with the results obtained for other species. For example, the optimal PVS2 treatment time for *Clinopodium odorum* shoot tip cryopreservation was 60 min at 0 °C using the V cryo-plate procedure [35]. However, the exposure time of the samples must be optimized for each species to assure tissue protection, e.g., the optimal exposure time to PVS2 at 0 °C was 30 min for blackberry apices and between 10 min and 30 min for cherry plums shoot tips [36], while in *Limonium serotinum* shoot tip regeneration was highest after PVS2 treatment for 30 min at 0 °C [37]. For two potato cultivars cryopreserved by the droplet-vitrification procedure, exposure to PVS2 for 40 min and 50 min at 0 °C gave the highest recovery rates, moreover apical buds responded better than axillary buds [38]. Irrespective of the vitrification solution used, the exposure time and temperature condition are fundamental and must be accurately determined depending on the plant material since some solutions can be toxic and even slight changes in treatment duration can have a dramatic impact on recovery [19].

The type of explant selected is also an important parameter for successful plant cryopreservation, in addition to size, cellular composition, physiological state and growth phase, which increase the probability of a positive response to various treatments before immersion in LN [9]. According to our cryopreservation data analysis, it seems that cryopreservation conditions proved to be more suitable for Stevia apical shoot tips than for axillary shoot tips. AST response is better for regrowth and in vitro subcultures, especially when a PVS2 exposure period of 60 min + LN was applied. The PVS2-treated AST had a better osmoprotective effect respect to AxST, both in DV (80% and 50%, respectively) and in V cryo-plate, (93% and 67%). In additional, it also showed the higher sensitivity of AxST compared to AST.

The DV procedure [19] is a method obtained by combining the vitrification procedure with the droplet-freezing technique developed by Kartha et al. [39] in which explants are placed in minuscule droplets of vitrification solution on aluminium foil strips, whereas the V cryo-plate procedure [20,40] combines the encapsulation and droplet-vitrification techniques, in which explants are placed on an aluminium cryo-plate and embedded in a thin layer of calcium alginate gel.

The two cryopreservation techniques (DV and V cryo-plate), applied on *S. rebaudiana* explants share the common trait of achieving higher cooling and warming rates compared to other vitrification-based procedures, because the explants placed on aluminium foils or cryo-plates, which are materials with high thermal conductivity, come into direct contact with liquid nitrogen during cooling and with the washing solution during warming [41]. Ultra-rapid cooling is more easily achieved using aluminium foil strips or aluminium cryo-plates rather than cryovials for immersing the explants directly into liquid nitrogen. These upgrades, combined with the optimal PVS2 exposure time, increased the chances of achieving a vitrified state during freezing in Stevia, resulting in superior regrowth after cryopreservation using the DV and V cryo-plate methods compared to results obtained by Shatnawi et coll. [7].

With respect to previous cryogenic methodologies, the simplicity is among the main advantages of using DV, moreover the technique can be successfully replicated by the technical staff [23]. The V cryo-plate method appears to be very systematic and time saving [42]. Both procedures appear highly promising to use large scale.

The DV method was initially used for potato shoot tip cryopreservation [43], but only a few years later its potential usefulness compared to other cryopreservation techniques was reported for several species such as in *Musa* [44], *Rosa* spp. [45,46,47], potato [25,48], taro [49], *Lilium* [50], *Saccharum* spp. [51], *Thymus* spp. [34], *Mentha* spp. [52], and *Vitis* spp. [29,53,54]. According to our results, applying this method to Stevia, shoot regrowth percentages of 80% and 50% were observed for cryopreserved AST and AxST, respectively. Panis et al. [44] reported that in eight different genomic groups of *Musa* spp., droplet-vitrification procedure increased regrowth by 23-46% compared to standard vitrification. In our study this procedure resulted in an improvement of 23% compared to the traditional vitrification method used by Shatnawi et al. [7] considering apical shoot tips as explants. By histological observations conducted on cryopreserved potato and pineapple shoot tips, some authors [55,56], concluded that droplet-vitrification caused the least injury to the tissues, thus it led to a benefit, increasing the recovery of cryopreserved shoot tips.

The application of V cryo-plate method has been reported for a wide range of species: strawberry [40], Dalmatian chrysanthemum [20], mint [42], mulberry [57], carnation [58], blueberry [59], mat rush [60], and sugarcane [61]. The satisfactory regrowth rates of cryopreserved potato shoot tips (96.7%) in V cryo-plate proved that it is a useful strategy for preserving valuable potato germplasm [62]. Recently, the V cryo-plate method has been successfully applied to grapevine germplasm [63].

Some authors have reported that the cryo-plate method is a user-friendly procedure that minimizes the risk of shoot injury, and permits high and rapid cooling and warming rates of treated materials, which improves recovery [21,59,64,65]. One of the various advantages of using the V cryo-plate procedure is that the explants are attached to the cryo-plates in alginate gel during the whole procedure, including thawing. Handling the explants throughout the procedure is very quick and easy because only the cryo-plates are manipulated, therefore all laboratory personnel can carry out this procedure once they are practiced at mounting the explants on the cryo-plates [20].

The V cryo-plate procedure increased the effectiveness of cryopreservation for *S. rebaudiana*, thus resulting in superior regrowth, 93% in AST and 67% in AxST, compared to the vitrification procedure used by Shatnawi et al. [7], who obtained a 68% shoot regrowth rate. Moreover, the growth of the shoots recovered from V cryo-plate procedure, during the subcultures was very dynamic and some evaluated parameters were slightly higher than the control group, especially for shoots from AST treated with PVS2 + LN for 60 min. However, after the second subculture, no significant differences were observed between the explant types tested and the control shoots, thus proving the effectiveness of the procedure.

In conclusion, Stevia responded well to conservation in LN and applying innovative approaches enabled us to increase the regrowth rate following cryopreservation, using both DV and V cryo-plate. It demonstrated that these techniques are efficient for the long-term storage of *S. rebaudiana*.

## 4. Materials and Methods

### 4.1. Plant Material and Culture Conditions

*S. rebaudiana* shoots (4–5 cm in length) were cultured on 100 mL of Murashige and Skoog (MS) [66] medium with 1 mg L^−1^ indole-3-butyric acid (IBA), 0.1 M sucrose and 7 g L^−1^ plant agar, at pH 5.8 in glass culture vessels (500 mL), with 20 shoots per vessel. The shoot cultures were maintained at 23 ± 1 °C under a 16 h photoperiod (40 μmol m^−2^ s^−1^). The shoots were subcultured monthly on fresh medium of the same composition (standard culture conditions).

### 4.2. Cold Hardening of In Vitro Shoot Cultures and Explant Preculture

*S. rebaudiana* shoot cultures (~4 cm in length with leaves) were transferred to 4 °C in low intensity light (25 μmol m^−2^ s^−1^) 7 days after the last subculture and maintained in hardening for 2 weeks before conducting cryopreservation experiments. Following preconditioning, apical shoots tips and axillary shoot tips, (explants with size of 1 mm to 1.5 mm for DV and 1 mm to 1.5 mm × 1 mm for V cryo-plate), were excised aseptically and placed in Petri dishes (9 cm Ø) containing hormone-free MS medium supplemented with 0.5 M sucrose at pH 5.8 and precultured for 48 h at 4 °C in darkness. Then, they were placed in loading solution (LS: MS liquid medium containing 2.0 M glycerol and 0.4 M sucrose at pH 5.8) [67] for 20 min at room temperature.

### 4.3. Evaluation of the Explant Tolerance to PVS2

In order to evaluate the effect of exposure duration to PVS2 solution (PVS2 contains 0.4 M sucrose, 30% (*w*/*v*) glycerol, 15% (*w*/*v*) ethylene glycol and 15% (*w*/*v*) dimethylsulfoxide in liquid MS medium, at pH 5.8) [17], the explants were incubated on sterilized PVS2 for 20, 30, 60, 90, or 120 min at 0 °C and 25 °C. The explants were then rinsed with Washing Solution (WS: liquid MS medium, containing 1.2 M sucrose [17] at 25 °C for 20 min and plated on proliferation medium (MS medium with 1 mg IBA, 0.1 M sucrose, 7 g L^−1^ plant agar, pH 5.8) for recovery under standard conditions.

### 4.4. Cryopreservation of S. rebaudiana Explants

The DV and V cryo-plate procedures were applied to the apical shoot tips and axillary shoot tips of *S. rebaudiana* (Figure 2).

#### 4.4.1. Droplet-Vitrification (DV) Procedure

The apical shoot tips (AST) and axillary shoot tips (AxST) were kept in LS at room temperature for 20 min and then placed into droplets of PVS2 (4 μL to 5 μL; each drop containing one explant) on sterilised aluminium foil strips (~6 mm × 25 mm, Figure 2a,b) in an open Petri dish at 0 °C and maintained under these conditions for 20, 30, or 60 min. Following PVS2 exposure, the aluminium foil strips with the explants were immersed into 2 mL Nalgene^®^ cryovials filled with liquid nitrogen (Figure 2c) and then plunged into LN for at least 1 h. For thawing, the frozen aluminium foils were removed from the cryovial and immersed immediately into Washing Solution at room temperature for 20 min (Figure 2d). Explants were placed on hormone-free MS and kept in the dark for at 22 ± 1 °C for 24 h and then transferred to standard culture conditions for recovery. The control group was composed of explants without any treatment.

#### 4.4.2. V Cryo-Plate Procedure

The aluminium cryo-plates (7 mm × 37 mm × 0.5 mm) with 12 wells (Ø 1.5 mm, depth 0.75 mm) were used. Droplets of approximately 2 μL of sodium alginate solution (the solution contains 2% (*w*/*v*) sodium alginate (Carlo Erba, Cornaredo, Milan, Italy; medium viscosity) in calcium-free MS basal medium with 0.4 M sucrose, at pH 5.8) were poured into the cryo-plate wells (Figure 2e). The explants were placed individually into each well with a scalpel blade and fully immersed in one drop of sodium alginate solution (Figure 2f). The calcium chloride solution (0.1 M calcium chloride in MS medium with 0.4 M sucrose, at pH 5.8) was poured dropwise onto the section of the cryo-plates until the explants were covered and then left for 15 min to achieve complete polymerization. The excess calcium solution was removed by sucking it with a micropipette. The cryo-plates with explants were placed in LS for 20 min at room temperature, the LS was then removed and the cryo-plates were filled with PVS 2 for 20, 30, 60, min at 0 °C. After each PVS2 treatment, the cryo-plates were transferred into 2 mL Nalgene^®^ cryovials placed in LN (Figure 2g), and then plunged directly into LN for at least 1 h. The cryo-plates with cryopreserved explants were thawed by immersing them in 2 mL of WS solution for 15 min. For recovery, the explants were placed on MS proliferation medium, in the dark for 24 h and then transferred to the light conditions described above. The control group was composed of explants without any treatment.

### 4.5. Data Collection and Statistical Analysis 

In order to evaluate PVS2 incubation times, the explant regrowth percentage was determined 4 weeks after the treatments, while for cryopreservation experiments, post-thaw survival rate data (i.e., percentage of explants that maintained their green colour and vigour) were recorded 7 days after transfer to proliferation medium, and the regrowth rate (the percentage of explants forming shoots ≥1cm) was recorded after 28 days. Each experimental treatment was replicated three times with each replication containing 10 shoot tips and nodal segments in the DV procedure and with 10 shoot tips and 15 nodal segments in the V cryo-plate procedure. For both procedures the following biometric parameters were recorded from regenerated cryopreserved shoots after the first subculture (28 days): (i) mean number of shoots, (ii) mean shoot length, and (iii) percentage of explants with shoots. In V cryo-plate procedure the biometric observations also involved the second subculture (28 days) with 3 replicates, 10 explants per each replicate. The data collected were subjected to one-way analysis of variance (ANOVA) followed by the Bonferroni post hoc test to determine the significance level between means at 95% confidence level (*p* ≤ 0.05). The percentage data were transformed in Arcsine before applying one-way ANOVA. The data were statistically analyzed using Statgraphics Centurion XVI (Stat Point, Inc., Herndon, VA, USA).

## Figures and Tables

**Table 1:** Droplet-vitrification procedure: effect of different PVS2 exposure times on survival and regrowth of apical shoot tips or axillary shoot tips after cryopreservation. Control (without any treatment).

Treatment	^1^ Survival Percentage (7 days)		Regrowth Percentage (28 days)	
	Apical Shoot Tips	Axillary Shoot Tips	Apical Shoot Tips	Axillary Shoot Tips
Control	100.0 a	93.0 a	93.0 a	93.0 a
PVS2 20 min + LN	6.0 c	0.0 c	0.0 d	0.0 c
PVS2 30 min + LN	46.6 bc	50.0 b	43.0 c	46.6 b
PVS2 60 min + LN	87.0 ab	50.0 b	80.0 ab	50.0 b

^1^ Statistical analysis in each column was performed by ANOVA. Data followed by different letters are significantly different at *p* ≤ 0.05 by Bonferroni’s test.

**Table 2:** First subculture after droplet-vitrification procedure: morphological parameters of *S. rebaudiana* shoots, from control and from cryopreserved apical shoot tips and axillary shoot tips. (Means ± SD).

Treatment	^1^ Shoot Length (cm)		Shoots/Explants (n°)		Explants with Shoot (%)	
	Apical Shoot Tips	Axillary Shoot Tips	Apical Shoot Tips	Axillary Shoot Tips	Apical Shoot Tips	Axillary Shoot Tips
Control	4.30 ± 0.48 ab	4.21 ± 0.64 a	0.96 ± 0.63 a	0.89 ± 0.73 a	78.0 a	68.0 a
PVS2 30 min + LN	4.00 ± 0.39 b	3.64 ± 0.44 b	0.84 ± 0.55 a	0.21 ± 0.42 b	77.0 a	21.0 b
PVS2 60 min + LN	4.90 ± 0.44 a	4.03 ± 0.39 ab	0.91 ± 0.58 a	0.80 ± 0.56 a	79.0 a	73.0 a

^1^ Statistical analysis in each column was performed by ANOVA. Data followed by different letters are significantly different at *p* ≤ 0.05 by Bonferroni’s test.

**Table 3:** V cryo-plate procedure: effect of different PVS2 exposure times on survival and regrowth of apical shoot tips or axillary shoot tips after cryopreservation. Control (without any treatment).

Treatment	^1^ Survival Percentage (7 days)		Regrowth Percentage (28 days)	
	Apical Shoot Tips	Axillary Shoot Tips	Apical Shoot Tips	Axillary Shoot Tips
Control	97.0 a	95.0 a	93.0 a	93.0 a
PVS2 20 min + LN	10.0 b	0.0 c	3.0 c	0.0 c
PVS2 30 min + LN	87.0 a	64.0 b	70.0 b	46.0 b
PVS2 60 min + LN	93.0 a	69.0 b	93.0 a	67.0 ab

^1^ Statistical analysis in each column was performed by ANOVA, Data followed by different letters are significantly different at *p* ≤ 0.05 by Bonferroni’s test.

**Table 4:** First subculture after V cryo-plate procedure: morphological parameters of *S. rebaudiana* shoots, from control and from cryopreserved apical shoot tips and axillary shoot tips. (Means ± SD).

Treatment	^1^ Shoot Length (cm)		Shoots/Explants (n°)		Explants with Shoot (%)	
	Apical Shoot Tips	Axillary Shoot Tips	Apical Shoot Tips	Axillary Shoot Tips	Apical Shoot Tips	Axillary Shoot Tips
Control	4.52 ± 0.70 a	4.16 ± 0.37 a	1.00 ± 0.74 a	0.97 ± 0.75 a	78.0 a	71.4 a
PVS2 30 min + LN	4.00 ± 0.51 b	3.95 ± 0.42 a	0.90 ± 0.57 a	0.81 ± 0.70 a	77.0 a	66.6 a
PVS2 60 min + LN	4.37 ± 0.78 a	4.20 ± 0.48 a	0.93 ± 0.60 a	0.73 ± 0.52 a	78.6 a	70.0 a

^1^ Statistical analysis in each column was performed by ANOVA. Data followed by different letters are significantly different at *p* ≤ 0.05 by Bonferroni’s test.

**Table 5:** Second subculture after V cryo-plate procedure: morphological parameters of *S. rebaudiana* shoots, from control and from cryopreserved apical shoot tips and axillary shoot tips. (Means ± SD).

Treatment	^1^ Shoot Length (cm)		Shoots/Explants (n°)		Explants with Shoot (%)	
	Apical Shoot Tips	Axillary Shoot Tips	Apical Shoot Tips	Axillary Shoot Tips	Apical Shoot Tips	Axillary Shoot Tips
Control	6.69 ± 0.98 a	6.52 ± 0.88 a	1.50 ± 0.68 a	1.43 ± 0.81 a	100.0 a	93.3 a
PVS2 30 min + LN	7.19 ± 1.00 a	6.43 ± 1.10 a	1.53 ± 0.81 a	1.40 ± 0.81 a	96.6 a	83.0 a
PVS2 60 min + LN	7.21 ± 1.40 a	6.44 ± 1.50 a	1.56 ± 0.96 a	1.40 ± 0.96 a	93.3 a	83.0 a

^1^ Statistical analysis in each column was performed by ANOVA, Data followed by same letters indicate no significant difference at *p* ≤ 0.05 by Bonferroni’s test.

## Data Availability

The data presented in this study are available on request from the corresponding author.
